# Seymour Benzer 1921–2007 The Man Who Took Us from Genes to Behaviour

**DOI:** 10.1371/journal.pbio.0060041

**Published:** 2008-02-12

**Authors:** William A Harris

## Abstract

Seymour Benzer died suddenly in December 2007. This piece pays tribute to Seymour Benzer's pioneering work in solid state physics, molecular biology, and neurogenetics.

Seymour Benzer was born in 1921 in the South Bronx, New York, the son of Polish Jewish immigrants. He was the only boy in a family that included his three sisters. His friend from later years, the phage biologist Jean Weigle, called Seymour the “egg with two yellows”, an old European expression for a rare event. He went to public schools in Brooklyn like any normal New York City kid, but everything changed when, at 13, a relative gave him a microscope for his Bar Mitzvah. “And that”, Seymour said, “opened up the whole world” [1]. He looked at everything he could find under the microscope, including flies—never imagining the remarkable discoveries he would later make about the way their brains worked.

With a Regents Scholarship to Brooklyn College in 1938, Seymour became the first in his family to go to college, where he studied physics. At college, Seymour met Dorothy (Dotty) Vlosky, a nursing student, and married her in 1942. Their wedding immediately preceded the couple's departure to Lafayette, Indiana, where Seymour was to continue his career as a graduate student in physics at Purdue. “We left the people dancing while we went to catch a train to Indiana”, Seymour said. “That was our honeymoon” [1].

At Purdue, Seymour joined the team of Karl Lark-Horovitz, which was then trying to find ways to make germanium semiconductors more reliable for radar, an important project during the war. At Purdue, Seymour came of age as a most remarkable scientist. He found that germanium crystals with trace amounts of tin were excellent rectifiers: conducting currents freely in one direction but resisting reverse flow without burning out even when sustained back voltages of more than 100 volts were applied [2]. Several industrial laboratories went into commercial production after the war using Seymour's patents. In 1948, a year after Seymour's thesis defence, Walter Brattain, John Bardeen, and William Shockley used these properties of germanium crystals to develop the first transistors at Bell Labs, for which they won the Nobel Prize. At the Bell Labs celebration, which Seymour attended, the developers grabbed him and said, “You should have done this!” But by that time, Seymour had already turned his attention to biology.

The emerging area of molecular genetics fascinated Seymour, and Lark-Horovitz was tremendously supportive of Seymour's desire to move into biology. Though Seymour had good job prospects in several physics departments, Lark-Horovitz offered him an Assistant Professorship at Purdue and, helping him make the transition to biology, granted Seymour an immediate leave-of-absence to begin postdoctoral research in phage genetics. This leave, initially intended to be for one year, stretched to two, then three, and finally four as Seymour's fellowships were extended and re-extended, though the Dean of Science at Purdue was getting ready to fire him for continuous absence. This is a period of his life about which Seymour used to reminisce with great fondness to his later students and post-docs, as it was during those years that he and Dotty formed great friendships with many of the historic figures of the early days of molecular biology, such as Max Delbruck and his colleagues at the California Institute of Technology (Caltech), where Seymour spent two years, and André Lwoff and his colleagues at the Pasteur Institute, where Seymour spent another year. With additional sojourns at Oak Ridge, Tennessee, and Cold Spring Harbor, New York, this was also the period when Seymour got the best possible basic training in phage genetics.

When he eventually did return to Purdue in 1952, it took Seymour little more than a year before he found a problem that he could really sink his teeth into. He discovered a way to explore the physical nature of the gene. This period of Seymour's research actually starts four decades earlier, when Alfred Sturtevant used the small fruit fly Drosophila to show that genetic factors map in a colinear array along chromosomes, based on the principle that the lower the frequency of recombination between them, the closer together the factors must be. Herman Muller incorporated this information to come up with the concept of a “gene”, a term he coined for a mutable, heritable unit of function that can be separated from other such units by recombination. But what, physically, was a gene? Was it made purely of nucleic acids, or was it part protein? Was it simply a linear stretch of DNA, or was it a globular bead?

By what in retrospect seems clearly a case of a prepared mind recognising the significance of an accidental finding, Seymour found that a gene called *r* (for *rapid lysis*) in the bacteriophage T4 would allow him to answer these questions. One day, while preparing for an upcoming teaching session, Seymour discovered that *rII* mutants failed to grow on a particular strain of bacteria that he had used. He recounted the fateful moment during an interview for the Caltech Oral History Project: “Well, at first I thought I made a mistake. I thought I had forgotten to put the phage on there. *Dummkopf,* do it again! I did it again and saw the same phenomenon. So I immediately realized—a Eureka moment—that this was a system in which I could do very fine genetic mapping!” [1]. (See [3] for a somewhat different view on this “Eureka moment”.)

He could infect this bacterial strain with two different *r* mutants. The *r* mutants themselves would produce no plaques, but if in any of the progeny there was a recombination between these two different mutations, that could produce a wild-type phage, which would produce a plaque on this bacterial strain. Seymour calculated that in his system he could use high enough titres of viruses to see extraordinarily low recombination frequencies. Using reasonable estimates of chromosome length (about 200,000 nucleotides), Seymour found that he had enough resolving power to separate mutations, even if the distance between them was less than the diameter of an electron! So he began collecting and making *rII* mutants and mapping them against each other, and with each round of experiments he went deeper into the fine structure of the gene. In the end, he had mapped about 20,000 mutants and deletions! The result of all this was that the *rII* gene could be represented by a colinear stretch of about 1,000 recombination units, each the size of a single nucleotide. The recombination map that Sturtevant had started almost half a century earlier, Seymour thus “ran into the ground”. This outstanding and beautiful work, published half a century ago, gave physical meaning to the gene. Frederick Holmes, in his book documenting Seymour's work on the gene, said that Seymour was the scientist who “more than any other single individual enabled geneticists to adapt to the molecular age” [3].

Yet this was not his only contribution to molecular biology. For example, he helped elucidate codon degeneracy by showing that distinct tRNA species (with different anticodons) can carry the same amino acid. But his days as molecular biologist were numbered! One day he received a letter from Delbruck, who complained that he was getting bored with Seymour's papers and wasn't going to read any more of them. (Delbruck was hard to please!) Seymour nevertheless took Delbruck's message to heart and decided to work on something new. The area of behaviour fascinated him, particularly because his two daughters had such different personalities. “I got interested in this general problem of personality and behavior—how much is genetics and how much is environment? And how do you study such a problem?” In 1966, he took a sabbatical from Purdue to begin this quest in Roger Sperry's lab at Caltech. During this year, Seymour settled on Drosophila as a research subject, for reasons he frequently explained thus: “If you're doing genetics, it's important to work with an organism where you can work on populations, because if you run a rat through a maze over and over again, it takes weeks to get any significant amount of data that would be statistically significant. But if you have a bunch of flies, they all have the same genotype, and when you run them through a maze, you immediately get to do hundreds of flies at once” [1].

Seymour accepted a position as a Professor of Biology at Caltech the following year. One of the first behaviours that he studied was phototaxis. He found that when flies are banged to the bottom of a test tube, they run like crazy to the light, a phenomenon known as fast phototaxis. Single flies, to Seymour, represented molecules or quanta of behaviour, and so he made a counter-current machine for flies based on the principles he and colleagues had used to separate different tRNA molecules. With this new machine, he could quickly separate flies that ran to the light from those that didn't, and run a new trial to separate them further. So in two minutes he had as much statistical information as it would have taken one several months to get with rats. He started feeding flies mutagens and then picking phototaxis mutants. Many mutants, though they appeared healthy, did not run to the light. Some of these, Seymour found, were blind because their photoreceptors did not transduce light, but others had defects at higher brain levels. Interestingly, he also found mutants that went into fits and others that were paralysed by being banged to the bottom of the tube. Using a variety of ingenious screens, Seymour and his quickly growing “neurogenetics” group found a whole spectrum of behaviours that could be changed by altering genes. There were learning mutants, optomotor mutants, paralytic mutants, hyperexcitable mutants, mutants that dropped dead because their brains degenerated early, homosexual mutants, etc. When his mother heard that Seymour was starting to work on the fly brain, her first reaction, Seymour recalled during the Caltech interviews, was, “From this you can make a living?” And then she took Seymour's wife aside and said, “Tell me, Dotty, if Seymour's going to examine the brain of a fly, don't you think we should have his brain examined?” [1].

His mother wasn't the only one who questioned his sanity. Many scientists, including some of his old molecular biology friends, Sydney Brenner and Gunther Stent in particular, and a large part of the neuroscience community, were highly sceptical of Seymour's approach. How was one going to find out anything about how the brain works by studying mutants? Was he foolish enough to imagine that it was going to be something simple like “one gene—one behaviour?” But Seymour was undaunted. He liked to ask what he referred to as “stupid questions”, because he believed that if you asked very naïve questions, you often found surprising answers. Such was the case when, with his graduate student Ron Kanopka, he started to look for mutants that affected circadian rhythms. Kanopka quickly identified the *period* gene via three alleles: *per* ^short^, which made the flies have a 19-hour rhythm instead of their normal 24-hour one, *per* ^long^, which gave mutants a 29-hour rhythm, and *per* ^0^, which was arrhythmic. When he told Delbruck about isolating these behavioural mutants, in a story Seymour loved to recite, Delbruck told him it was impossible. To which Seymour responded, “But, Max, we found the gene, we've already done it!” Still Delbruck insisted that it was impossible and told Seymour, “I don't believe a word of it” [3,4]. Though one of Seymour's greatest supporters, Delbruck *was* hard to please. This story is quintessential Seymour—always a maverick, trusting his instincts even in the face of formidable doubters—and marks the beginning of his remarkable foray into neurogenetics—the field he founded.

Those of us who worked in his lab at the time were inspired by his creativity, his modesty, his weird sense of humour, his exotic tastes in food, but most of all by the enormous sense of fun and possibility he exuded as he began to explore this new field. Many of us fondly remember sitting around the steel-topped table in Seymour's lunchroom, where the whole lab would take our packed lunches each day. Seymour, a clock mutant himself, usually ambled into the lab just before lunch, often bringing in something unbelievably disgusting for us to taste such as rotten fish or chocolate-coated grubs, and often he would have a story about something very strange or macabre he had recently done, like attending the Hollywood funeral of a famous actor's dog. Frequently, a well-known scientist would visit the lunchroom, and Seymour would ask one of us to say something about our research. This would launch an animated discussion that sometimes lasted most of the afternoon. The lab would fire up in the evening, which is when we'd really start beavering away. If you stumbled off to bed at midnight, you'd see that the light was still on in Seymour's lab/office, where he would potter away at his own experiments until the early hours.

His work, and that of his still growing posse of scientific disciples, eventually convinced even the most critical sceptics that this genetic approach to behaviour was mining a rich seam of extraordinary information about neural development, cellular and molecular neurobiology, circuit function, learning and memory formation, and even cognitive function.

It is not surprising that Seymour won several of the grand prizes in biology, but what is striking is that they came from so many disciplines. He won the Gairdner Foundation International Award twice (first for molecular biology in 1964 and then for neurogenetics in 2004), the Rosenstiel Award and the Thomas Hunt Morgan Medal for genetics (1986, 1989), the Ralph W. Gerard Prize in Neuroscience (1989), the Wolf Prize in Medicine (1991), and the March of Dimes Prize in Developmental Biology (2002), to name only some. He also won the Albert Lasker Award for Basic Medical Research (1971), the Crafoord Prize (1993) from the Royal Swedish Academy of Sciences “for work not covered by the Nobel”, and the Albany Medical Center Prize (2006), which is often called the “American Nobel Prize in Physiology or Medicine”. But he never received the Nobel itself, either for his extraordinary work on the gene or for his seminal work in neurogenetics. And because of that, Seymour said, tongue-in-cheek, his mother regarded him as a failure. “These other prizes don't mean anything to the neighbors”, she told him [1].

Several years after Dotty died, Seymour married Carol Miller, a neuropathologist at the University of California, Los Angeles. Seymour had often admitted to colleagues that he wanted to work on humans because they show such interesting and bizarre behaviours. And although he continued to study Drosophila, his association with Carol led him to do more medically relevant biology, such as Drosophila models of neurogenerative diseases. In recent years, he and his colleagues found several mutants that dramatically extended the average lifespan of Drosophila, the first of which he called “Methuselah”. Many of us hoped that Seymour, with his perpetual sense of fun and naïve way of approaching problems, might discover a secret to aging and live as long as Methuselah. But it was not to be, as he suddenly died of a stroke at 86. It is hard for those of us who were strongly influenced by him to imagine Seymour no longer alive because we feel his intellectual and human presence in our work each day. When he died, so died one of the great scientists of our age. He discovered so much and, in so doing, opened up so much more to discover.

**Figure:**
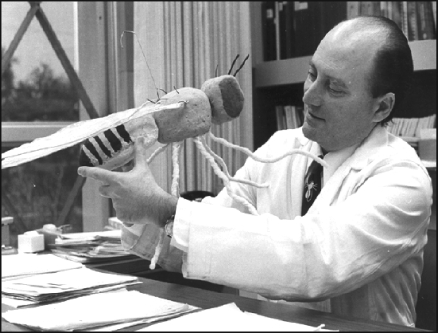
Seymour Benzer in his office at Caltech in 1974 with a big model of Drosophila. He had a great deal of respect for an animal that not only can perform many sophisticated behaviours that humans do—such as learning, courting, and keeping time—but can also walk on the ceiling and fly.
